# Postoperative Complications in Emergency Surgeries at a Referral Hospital in Eastern Venezuela

**DOI:** 10.7759/cureus.59805

**Published:** 2024-05-07

**Authors:** Victor Castañeda-Marquez, Yeisson Rivero-Moreno, Enrique Avila-Liendo, Gabriel Gonzalez-Quinde, Wilson Garcia-Cazorla, Georcimar Mendez-Meneses, Yoalkris E Salcedo, Tamara Rodriguez-Rugel, Jackner Antigua-Herrera, Miguel Rivas-Perez, Silvia Agudelo-Mendoza, Cesar Estrella-Gaibor

**Affiliations:** 1 Department of Surgery, Universidad de Oriente, Nucleo Anzoategui, Barcelona, VEN; 2 Department of Surgery, Hospital Leon Becerra Camacho, Milagro, ECU; 3 Department of Surgery, Universidad de Cuenca, Cuenca, ECU; 4 Department of Surgery, Universidad Iberoamericana, Santo Domingo, DOM; 5 Department of Surgery, Universidad Católica de Santiago de Guayaquil, Guayaquil, ECU; 6 Department of Surgery, Instituto Tecnológico de Santo Domingo, Santo Domingo, DOM; 7 Department of Surgery, Universidad Del Rosario, Bogota, COL; 8 Department of Surgery, Ministerio de Salud Pública, Hospital Esmeraldas sur Delfina Torres de Concha, Quito, ECU

**Keywords:** trauma, venezuela, surgical site infection, emergency surgery, postoperative complication

## Abstract

Background

Postoperative complications (POC) are undesirable consequences of surgery and are a major area of concern adversely affecting the quality of surgical care and patient safety. Emergency surgery has been observed to have a higher incidence of different POC. The analysis of POC is of great importance due to their impact on the quality of life of patients and because they have become an indicator to measure the quality of hospital services.

Objective

This study aimed to describe the POC of emergency surgeries in patients from the general surgery department of a referral hospital in eastern Venezuela.

Methodology

A cross-sectional retrospective study was conducted, including patients undergoing emergency surgery at "Dr. Luis Razetti" University Hospital, Barcelona, Venezuela, between November 2022 and May 2023.

Results

Medical records of 178 patients were analyzed. Most were male (53.7%), with an average age of 34.98 and a standard deviation of 18.2 years. POC was registered in 28 (15.7%) patients, with surgical site infection being the most common in 21 (39.62%) patients. Those over 65 years old (21.4% vs. 6.4%, p=0.013), patients with a history of hypertension (25% vs. 6.3%, p=0.002), hypoalbuminemia (100% vs. 43.8%, p=0.027), diagnosed with peritonitis due to hollow viscus perforation (21.4% vs. 6%, p=0.007), trauma (25% vs. 9.3%, p=0.018), and those with a midline incision (75% vs. 31.3%, p<0.001) had a higher frequency of POC. There was a mortality rate of 2.8% with no significant difference based on the development of POC.

Conclusion

POC represents a significant cause of morbidity and mortality in patients undergoing emergency surgeries. The studied sample showed a similar frequency of POC compared to previous reports but with lower mortality. Complications were associated with higher frequencies of hypertension, midline approach, hypoalbuminemia, and emergency surgery for peritonitis due to hollow viscus perforation and trauma.

## Introduction

Emergency surgery can be defined as surgery that is required to deal with an acute threat to life, organ, limb, or tissue caused by external trauma, acute disease process, acute exacerbation of a chronic disease process, or complication of a surgical or other interventional procedure [1].

Postoperative complications (POC) are undesirable consequences of surgery and are a major area of concern adversely affecting the quality of surgical care and patient safety. These range from seemingly minor incidents that resolve without any harm to more serious incidents that may pose a threat to life, need multiple interventions, prolong hospital stay and costs, and may at times cause disability or death [2].

Emergency surgery has been observed to have a higher incidence of different POCs such as surgical site infection (SSI), pneumonia, myocardial infarction, major bleeding, and stroke with higher subsequent mortality [3].

It is known that significant POC is already common and occurs in around 20% of patients, although the reported incidence may depend on the detection methods used [4]. Complications come at a cost, financial and otherwise, to a range of stakeholders. Some studies on complications after abdominal surgery showed that costs doubled for minor complications and doubled again for major complications [4]. POC is a leading cause of long-term morbidity and mortality. Reported data suggest that 4.2 million deaths will occur within 30 days of surgery worldwide each year, half of which are in low- and middle-income countries [5].

In a study that evaluated nearly 1.5 million patients who underwent 115 types of gastroenterological surgeries, at least 14,000 patients died from POC per year [6].

The analysis of POC is of great importance due to their impact on the quality of life of patients and because they have become an indicator to measure the quality of hospital services. Various factors have been associated with the occurrence of POC, such as age, comorbidities, the quality of surgical technique, and postoperative care, among others [7].

In Latin America, there is a lack of multinational registries that describe in detail the incidence of POC, although efforts have been made to collect such information [5]. In Venezuela, few studies have been published on POC, despite the increasing demand for emergency abdominal surgery services, similar to other parts of the world. In the study by Palacios, POC was described in 152 patients undergoing emergency abdominal surgery at a referral hospital in the northwest region of the country [8].

Currently, there are no published studies on POC in patients undergoing emergency surgery in the eastern region of the country. Therefore, the objective of this study was to describe the POC in patients from the general surgery department of the "Dr. Luis Razetti" University Hospital in Barcelona, Venezuela.

## Materials and methods

A cross-sectional retrospective study was conducted. The study population consisted of patients who underwent any surgical intervention in the general surgery department of the "Dr. Luis Razetti" University Hospital, the university hospital for the School of Medicine at Universidad de Oriente, between November 2022 and May 2023. Patients over 12 years old who required emergency or urgent surgical resolution in the general surgery department of the mentioned center were included. Patients with incomplete medical records and those without data from the postoperative period were excluded. All patients who met these criteria were included in the study, as convenience sampling was used.

Through a detailed review of medical records including surgical notes, the following data were extracted from the study population namely age, sex, comorbidities, results of paraclinical studies (including leukocytes, hemoglobin, serum albumin, blood glucose, and serum creatinine), admission diagnosis, type of surgical approach, types of anesthesia, type of incision, use of drainage, use of vasoactive drugs, length of hospital stay, non-programmed re-intervention, and death. Our main study outcome was the frequency and type of POC. The classification of patients with anemia based on hemoglobin values was determined according to the reference values provided by the World Health Organization (WHO), considering normal hemoglobin levels to be 12-16 g/dL in women and 14-18 g/dL in men [9]. Leukocytosis was defined as a leukocyte count greater than 11,000 white blood cells per microliter and was expressed as a percentage of patients with leukocytosis. Normal albumin values were considered to be between 3.5 and 5.5 g/dL, glucose levels between 70 and 100 mg/dL, and creatinine levels between 0.7 and 1.3 mg/dL for men and 0.6 and 1.1 mg/dL for women [10,11]. The information corresponding to these variables was extracted from the medical records and entered into a Microsoft Excel datasheet (Microsoft Corporation, Redmond, United States).

Data were presented as mean and standard deviation (SD) for quantitative variables and percentages for qualitative variables. The association between qualitative variables was assessed using the chi-square test and the exact mid-p test, and the association between quantitative variables was assessed using the Student t-test. A p-value <0.05 was considered statistically significant. For statistical analysis and reporting of results, the IBM SPSS Statistics for Windows, Version 29 (Released 2021; IBM Corp., Armonk, New York, United States) and the OpenEpi version 3.01 online tool were utilized.

The present study was conducted following the norms and guidelines outlined in the STROBE statement and approved by the general surgery department and the Bioethics Committee of the "Dr. Luis Razetti" University Hospital under the Memorandum N° 120-HULR-2022.

## Results

**Figure 1:**
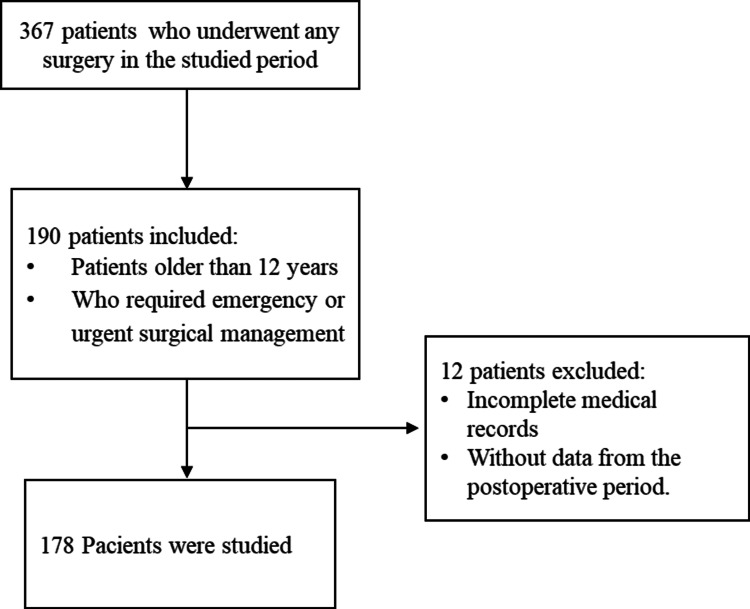
Patient selection flowchart

During the study period, 367 patients underwent surgery, of which 178 met the inclusion criteria. The details regarding patient selection are shown in Figure 1.

Most of the patients were male (53.7%), with an average age of 34.98 years and a SD of 18.2. The most numerous age group was patients between 20 and 59 years old (64.6%). The frequency of POC was higher in patients over 65 years old (21.4% vs. 6.4%, p=0.013). Details of the clinical and epidemiological characteristics of the evaluated patients are presented in Table 1.

**Table 1:** Clinical and epidemiological characteristics of patients undergoing emergency surgery based on postoperative complications HTA: hypertension; DM2: type 2 diabetes mellitus ^a^ Continuous data are shown as the mean±SD and categoric data as number (%) ^b^ Percentage base in the total of comorbidities reported ^c^ "Trauma" encompasses thoracoabdominal trauma, both open and closed, as well as injuries caused by firearm projectiles ^* ^Chi-square ^†^ Mid-p exact ^‡^ T-student Bold values are statistically significant. Normal laboratory test values: hemoglobin: females (12–16 g/dL), males (14–18 g/dL); leukocytes: leukocytosis (>11,000 white blood cells per microliter); albumin (3.5 to 5.5 g/dL); glucose (70 to 100 mg/dL); creatinine: males (0.7 to 1.3 mg/dL), females (0.6 to 1.1 mg/dL).

	Total (n=178)	Postoperative complications		p-values
Characteristics^a^		Yes (n=28)	No (n=150)	
Sex - Male	96 (53.9)	19 (67.9)	77 (51.3)	0.107^*^
Age (years)	34.98±18.2	43.07±21.27	33.47±17.24	0.100^‡^
Comorbidities^b^	-			
HTA	16 (9.3)	7 (25)	9 (6.3)	0.002^*^
DM2	6 (3.5)	1 (3.6)	5 (3.5)	0.979^*^
Asthma	4 (2.3)	-	4 (2.8)	-
Previous abdominal surgery	47 (27.5)	7 (25)	40 (28)	0.747^*^
Psychiatric disorders	2 (1.2)	-	2 (1.4)	-
Admission laboratory tests	-			
Leukocytosis (%)	67 (50.4)	9 (42.9)	58 (51.8)	0.453^*^
Hemoglobin (gr/dl)	12.28±2.2	11.42±3.15	12.44±1.95	0.051^‡^
Albumin (g/dl)	3.4±0.73	2.81±0.61	3.59±0.68	0.035^‡^
Glucose (mg/dl)	98.16±29.32	100.34±21.27	97.72±30.85	0.790^‡^
Creatinine (mg/dl)	0.98±0.78	0.82±0.25	1.02±0.87	0.396^‡^
Admission Diagnosis	-			
Acute appendicitis	81 (45.5)	2 (7.1)	79 (52.7)	<0.001^†^
Peritonitis due to hollow visceral perforation	15 (8.4)	6 (21.4)	9 (6)	0.007^*^
Acute cholecystitis	13 (7.3)	1 (3.6)	12 (8)	0.460^†^
Trauma^c^	21 (11.8)	7 (25)	14 (9.3)	0.018^*^
Intestinal obstruction due to neoplasms	13 (7.3)	4 (14.3)	6 (4.8)	0.063^†^

Within the 47 patients with a history of previous abdominal surgery, 22 (46.8%) were related to gynecological interventions, 7 (14.9%) to appendectomies, and 6 (12.77%) to umbilical hernia repairs.

The results of hemoglobin levels were obtained from 131 patients, 64 (48.9%) women and 67 (51.1%) men. Anemia was recorded in 19 (29.7%) women and 16 (23.9%) men (p=0.4528). It was found that the development of POC was higher among women with anemia (38.1% vs. 16.7%, p=0.029). Such association was not found in the group of men. Among patients with recorded serum albumin levels (n=21), hypoalbuminemia was recorded in 12 (57.1%). It was found that the average albumin levels were lower in those patients with POC; furthermore, when compared by categories, it was observed that the frequency of hypoalbuminemia was higher in that group (100% vs. 43.8%, p=0.027).

It was observed that patients with a diagnosis of acute appendicitis had a lower frequency of POC (p<0.001), while patients diagnosed with peritonitis due to hollow viscus perforation (p=0.007) and trauma (p=0.018) had a higher frequency of complications. Among the different types of trauma, the most common was firearm projectile trauma in 13 (61.9%) of the patients.

In Table 2, the surgical and postoperative period characteristics of the studied patients are detailed. It was observed that patients with a midline incision had a higher frequency of POC (p<0.001). Similarly, hospitalization days and the rate of reinterventions were higher in patients with POC (p<0.001). There was a mortality rate of 2.8% with no significant difference based on the development of POC.

**Table 2:** Surgical and postoperative period characteristics in patients undergoing emergency surgeries based on postoperative complications ^a^ Continuous data are shown as the mean±SD and categoric data as number (%) ^*^ Chi-square ^†^ Mid-p exact _‡_ T-Student Bold values are statistically significant.

-	Total (n=178)	Postoperative complications		p-values
Characteristics^a^		Yes (n=28)	No (n=150)	
Surgical approach type	-			
Open	160 (89.9)	26 (92.9)	134 (89.3)	0.570^*^
Laparoscopic	18 (10.1)	-	18 (12)	-
Anesthesia types	-			
Percutaneous	178 (100)	28 (100)	150 (100)	-
Inhalatory	163 (91.6)	27(96.4)	136 (90.7)	0.314^*^
Conductive	8 (4.5)	-	8 (5.3)	-
Infiltrative	178 (100)	28 (100)	150 (100)	-
Incision type	-			
Infraumbilical	13 (7.3)	1 (3.6)	12 (8)	0.408^†^
Supraumbilical	4 (2.2)	-	4 (2.7)	-
Midline	68 (38.2)	21 (75)	47 (31.3)	<0.001^*^
McBurney technique	44 (24.7)	1 (3.6)	43 (28.7)	0.016^†^
Kocher technique	11 (6.2)	-	11 (7.3)	-
Use of drain	4 (2.2)	2 (7.1)	2 (1.3)	0.057^†^
Use of vasoactive drugs	1 (0.6)	-	1 (0.7)	-
Hospitalization days	4.84±6.97	12.96±13.19	3.33±3.4	<0.001^‡^
Non-programmed re-intervention	24 (13.5)	22 (78.6)	2 (1.3)	<0.001^*^
Mortality	5 (2.8)	2 (7.1)	3 (2.0)	0.131^†^

The list and frequencies of the main POC are presented in Table 3. A total of 53 POC were recorded in 28 patients, representing a frequency of 15.7%. The most common complication was SSI in 21 patients (39.62%), of which 16 (64.7%) were superficial infections.

**Table 3:** Distribution of frequencies of main postoperative complications in patients undergoing emergency surgery SSI: surgical site infection

Complication	N (%)
SSI	21 (39.62)
Anastomotic leak	9 (16.98)
Evisceration	8 (15.09)
Fistulas	5 (9.43)
Hemorrhage	2 (3.77)
Incidental injury	2 (3.77)
Atelectasis	2 (3.77)
Urinary tract infection	1 (1.89)
Bilioma	1 (1.89)
Sepsis	1 (1.89)
Pneumonia	1 (1.89)

In patients with acute appendicitis, the frequency of SSI was lower (19% vs. 49%, p=0.009), while in patients diagnosed with peritonitis due to hollow viscus perforation upon admission (28.6% vs. 5.7%, p<0.001), and those with midline incisions (71.4% vs. 33.8%, p=0.001), there was a higher frequency of SSI.

## Discussion

The aim of this study was to describe POC in patients from a referral hospital in eastern Venezuela, finding such complications in less than one-sixth of the studied population, with SSI being the most frequent, and reporting that certain factors, such as hypertension, midline approach, and hypoalbuminemia, were associated with a higher frequency of POC.

The majority of the analyzed patients were male, similar to other studies focusing on patients undergoing emergency surgeries, such as that of Lepercq et al. in 2023 with over 1100 patients [12]. While this trend remains consistent across different studies, the mean age may vary depending on the patient cohort, in some cases with higher average ages [12,13] or similar to ours [14].

A retrospective review of the American College of Surgeons National Surgery Quality Improvement Program database (ACS NSQIP), conducted at 435 hospitals nationwide, reported that the odds ratios for experiencing any complication after urgent or emergency surgery were higher compared to elective surgery. Similarly, the odds ratios for mortality were higher in these types of procedures [15]. Therefore, it is essential to describe in detail these types of procedures and determine possible factors associated with a higher frequency of them.

In our study, the frequency of POC was 15.7%, similar to the range of values reported in different studies such as Ludbrook which placed it around 20% [4], or the study by Karna et al. with patients from India reporting an incidence of 22% [14]. Meanwhile, according to the ACS NSQIP report, the incidence of complications between urgent and emergency surgeries ranges from 12% to 14% [15].

According to the study by Dencker et al., evaluating the trends of POC rates over a seven-year period based on the NSQIP datasets, SSI was among the top three most common complications in the postoperative period [16]. Our study reported SSI as the most common POC (39%). However, these figures are higher than those reported in previous studies with patients similarly undergoing emergency surgery, such as Papadopoulos et al. reporting SSI in 18.7% of patients [17], or Jatoliya et al. with 26% [18]. This high frequency of SSI should be of concern because the ways to prevent them are widely known and accessible for application, and ideally, the trend should be that they occur less frequently, as found by Dencker et al. in the USA [16].

Some factors such as the presence of hypertension, hypoalbuminemia, emergency surgery for peritonitis due to hollow viscus perforation or trauma, as well as a midline incision approach, were more common in the group of patients with POC. Similarly, the study by Dharap et al. with patients undergoing emergency surgery in Mumbai reported a statistically significant association between the presence of comorbidities and the development of POC [2]. Other studies have also pointed out the higher morbidity and mortality in patients after intestinal perforation [19]. Similarly, previous studies have reported an association between hypoalbuminemia and the occurrence of POC [20].

It has been widely demonstrated that patients undergoing emergency surgery have a higher risk of complications and death compared to those undergoing elective procedures, but mortality rates vary among studies [15]. The study by Degu et al., with over 3000 patients, reported a mortality of 6.36% in patients undergoing emergency surgery [21]. Dharap et al. reported a mortality of 3.75% [2]. Meanwhile, Bainbridge et al. reported a 1.2% aggregate mortality for elective surgery and 10.1% for emergency surgery in a systematic review of studies from low- and middle-income countries [22]. However, our study reported a mortality rate of 2.8%, slightly lower than that reported in the aforementioned studies. The distribution and severity of the reported complications, the average age of the patients, and the timely management of such complications could contribute to lower mortality compared to other studies, despite reporting a similar frequency of POCs as found in the literature.

The conclusions of the present study should be interpreted within the following limitations. The sample size may have limited the ability to find some possible associations, such as in the case of mortality where few cases were recorded. However, this study is one of the few, to our knowledge, that examines POC in detail in patients undergoing emergency surgery in Venezuela, and the first in the eastern region of the country. Given the importance of studying POC, it would be advisable to conduct a prospective cohort study to determine the risk factors for POC specifically, thus enabling the implementation of corresponding preventive measures.

## Conclusions

POC represents a significant cause of morbidity and mortality in patients undergoing emergency surgeries. The studied sample showed a similar frequency of POC compared to other reports but with lower mortality. These complications were associated with higher frequencies of hypertension, midline approach, hypoalbuminemia, emergency surgery for peritonitis due to hollow viscus perforation, or trauma. SSI remains one of the leading causes of POC, particularly elevated in this study. Further prospective cohort studies are advisable to determine the risk factors for POC specifically, thus enabling the implementation of corresponding preventive measures.
